# Encapsulation protocol for fecal microbiota transplantation

**DOI:** 10.3389/fcimb.2024.1424376

**Published:** 2024-06-26

**Authors:** Dávid Sipos, Adorján Varga, Ágnes Kappéter, Bernadett Halda-Kiss, Péter Kása, Szilárd Pál, Béla Kocsis, Zoltán Péterfi

**Affiliations:** ^1^ 1^st^ Department of Internal Medicine – Department of Infectology, University of Pécs Clinical Centre, Pécs, Hungary; ^2^ Department of Medical Microbiology and Immunology, University of Pécs Medical School, Clinical Centre, Pécs, Hungary; ^3^ Institute of Pharmaceutical Technology and Biopharmacy, University of Pécs Faculty of Pharmacy, Pécs, Hungary

**Keywords:** *Clostridioides difficile*, *Clostridium difficile* infection, FMT, protocol, capsule, lyophilizate

## Abstract

**Introduction:**

*Clostridioides difficile* infections (CDI) continue to pose a challenge for clinicians. Fecal microbiota transplantation (FMT) is an effective treatment option in CDI. Furthermore, recent and ongoing studies suggest potential benefits of FMT in other diseases as well.

**Methods:**

We would like to present a novel protocol for encapsulation of lyophilized fecal material. Our method provides with better compliance as well as improved flexibility, storage and safety.

**Results:**

FMT was conducted in 28 patients with an overall success rate of 82,14% using apsules containing lyophilized stool. 16 of patients were given capsules with lessened bacteria counts. The success rate in this group was 93,75%.

**Discussion:**

The results highlight the still unanswered questions about the mechanism of action and contribute to a wider use of FMT in the clinical praxis and in research.

## 1 Introduction

Healthcare-associated infections are diseases that occur despite the work of health care workers or as a side effect of health care interventions. They impose a significant burden on patients and the healthcare system. *Clostridioides difficile* infection (CDI) is a typical example of this group of diseases.

Most often, *C. difficile* is unlikely to enter the body during hospital care. The bacteria are often carried by clinically symptomatic patients even before admission (Péterfi, 2015). It has also been shown to be an essential element of the intestinal flora of infants (Jangi and Lamont, 2010) and is not uncommon in the (even healthy) population without symptoms of CDI (Nagy et al., 2013).

Restoring colonization resistance with bactericidal or bacteriostatic drugs is a significant challenge. It can be restored by introducing healthy gut flora or even bacterial strains specific to healthy gut flora into the intestinal tract. It is recorded to have been a common procedure in ancient China (Zhang et al., 2012) and in 18th century Europe for the treatment of various gut-related pathological conditions (DePeters and George, 2014).

The whole gut flora is in fact a “finished product”, but this can also allow the transmission of pathogens (DeFilipp et al., 2019; Zellmer et al., 2021). It is also possible to introduce a known composition of bacterial strains, but the complexity of the gut flora makes it very difficult to determine the correct composition.

The main aim of this research was to improve the already proven effectiveness of fecal transplantation and, as part of this, to make it a more acceptable procedure for patients and easier to perform in clinical practice. For this purpose, we considered capsules filled with lyophilized stool to be the most suitable due to their ease of storage, transport and use. Sample preparation inevitably became more complex; therefore, the workflow was designed to fit in with the daily tasks of a normal working day.

### 1.1 Pathophysiology of CDI

*Clostridioides difficile* is resistant to commonly used surface disinfectants due to its spore-forming ability and is also tolerant to desiccation, allowing it to survive for extended periods on various surfaces (Kramer et al., 2006). Removal requires a sporicidal disinfectant and hand washing with soap (Cooper et al., 2016). It is also commonly found in the gut flora of asymptomatic individuals. Illness is typically caused when the colonization resistance of the intestinal flora is weakened. Elderly, immunosuppressed state, prolonged antibiotic use predicts more severe clinical symptoms.

Its spread between patients in a healthcare or nursing facility is of particular importance in the case of toxin-producing (toxin A, toxin B and binary toxin) strains, which cause a more severe course (Chandrasekaran and Lacy, 2017). Ribotype 027 produces 16 times more toxin A and 23 times more toxin B (Warny et al., 2005).

Toxin A is an enterotoxin that destroys cell-to-cell contacts, while toxin B and the binary toxin are cytotoxins, with toxin B targeting the actin filament that builds the cytoskeleton. Together, the toxins therefore induce symptoms by disrupting the integrity of the intestinal mucosa. The disease may be associated with mild intestinal symptoms, but also with painful, mucopurulent (rarely bloody) diarrhea, for example in pseudomembranous enterocolitis.

Persistent, asymptomatic *C. difficile* carriage is a protective factor for CDI. This is likely to be due to elevated serum levels of antibodies against toxins A and B. A similar protective effect has been observed in non-toxin-producing strains in both human and animal studies (Johnson et al., 2021).

Recurrence of the disease is also an unfavorable sign both for the remission of the current disease and for new recurrences. The frequency of recurrence of CDI and the time and cost of hospitalization places a heavy burden on the healthcare system (Ghantoji et al., 2010; Dubberke and Olsen, 2012).

### 1.2 Treatment of CDI

The conventional first-line treatment is metronidazole, with vancomycin used for more severe cases or relapses. Multiple relapses or a very severe course justifies the use of fidaxomicin (van Prehn et al., 2021). Other drug therapy options include nitazoxanide, rifaximin, tigecycline and teicoplanin and certain monoclonal antibodies (Wilcox et al., 2017).

Non-drug treatment is fecal transplantation, which rivals the efficacy of the most effective anti-CDI drugs (Vigvári et al., 2015). Recent publications have shown similarly reliable results with a concentrate of *Firmicutes* strain spores (SER-109).

#### 1.2.1 Antimicrobials

Metronidazole has bactericidal activity against anaerobic bacteria. It can be administered orally and parenterally. Due to its efficacy in mild to moderate CDI and their relatively low cost (Venugopal and Johnson, 2012) it is still in widespread use. According to the latest guidelines, they should only be used under specific circumstances.

Vancomycin inhibits bacterial cell wall synthesis. It is normally used as an agent in intravenous formulations. In CDI, it is dissolved in water and administered orally. An argument against its use in CDI is that it may cause the proliferation of resistant strains of other pathogens in the intestinal tract (e.g. *Enterococcus* and *Staphylococcus* strains) (Mathias et al., 2019).

Fidaxomicin has a lower MIC against *C. difficile* strains (including ribotype NAP1/B1/027) than metronidazole or vancomycin under *in vitro* conditions. Due to its narrow antibiotic spectrum, it is less disruptive to the diversity of the microbiome and thus allows rapid recovery of colonization resistance. It has been observed to reduce sporulation and toxin production. Its effect in reducing the incidence of recurrent CDI makes it cost-effective in the group of patients at highest risk of relapse (Mullane, 2014).

Tigecycline, an intravenously administered tetracycline derivative, is effective against a number of multi-drug resistant pathogens (Mathur et al., 2014).

The following antibiotics are not part of the ESCMID recommendation but have also been shown to be effective in the treatment of CDI.

Nitazoxanide is comparable to vancomycin in terms of efficacy against *C. difficile*. It is considered a possible choice in refractory or recurrent cases (Mathur et al., 2014).

Rifaximin is a gastrointestinal-selective antibiotic with broad-spectrum bactericidal activity, but only negligible effect on normal gut flora (Mathur et al., 2014).

Teicoplanin also shows good results, but its availability and high cost limit its use (Mathur et al., 2014).

Ridinilazole is a benzimidazole derivative. According to ongoing trials, its efficacy in CDI exceeds that of vancomycin (Vickers et al., 2017).

#### 1.2.2 Fecal microbiota transplant

Its use in the therapy of pseudomembranous colitis was first reported in 1958 (Eisenman et al., 1958) and has since been shown to be an effective and safe procedure in numerous studies (Kelly et al., 2014). It is currently part of the therapeutic protocol for recurrent CDI. The exact criteria for ideal donors are not clear. In principle, healthy individuals with no infectious disease based on their medical history and screening results are requested for donation. A general bacteriology, parasitology and *C. difficile* toxin testing of stool samples and screening of serum samples for HIV-1, HIV-2, EBV, CMV, hepatitis A, B, C, *Treponema pallidum subspecies pallidum* are routinely performed (Vigvári et al., 2015). SARS-Cov-2 virus RNA can be detected in stool long after recovery and protocols have been developed for screening donors (Ianiro et al., 2020; Nagy et al., 2020; Segal et al., 2020).

There are currently four different options for fecal transplantation. It can be performed rectally, through a nasogastric (NG) or a nasojejunal (NJ) tube. Samples prepared for this purpose can be stored frozen (up to -20°C) for later use (Youngster et al., 2014; Vigvári et al., 2015). Encapsulation is the fourth option. Among these, the use of frozen stool-filled capsules (Vigvári et al., 2019) and lyophilized inoculum-filled capsules (Varga et al., 2021; Varga et al., 2023) is possible. Case series studies have shown the efficacy of stool transplants in recurrent CDI, including the prevention of relapse after multiple relapses (Vigvári et al., 2015).

The microbiome is likely to play an important role in irritable bowel disease (Ford et al., 2020), obesity, metabolic syndrome and diabetes (Aron-Wisnewsky et al., 2021). The protocol of the European Society of Clinical Microbiology and Infectious Diseases (ESCMID, **Table 1**) recommends it for severe complicated and refractory infections (van Prehn et al., 2021).

**Table 1:** The protocol of the European Society of Clinical Microbiology and Infectious Diseases in CDI.

		Initial CDI	First recurrence	Further recurrences
**Standard of care (SoC)**	**1^st^**	Fidaxomicin 200 mg bid 10 days		FMT
	**2^nd^**	Vancomycin 125 mg qid 10 days		SoC + Bezlotoxumab
**High risk of recurrence**	**1^st^**	Fidaxomicin 200 mg bid 10 days		
	**2^nd^**	SoC + Bezlotoxumab		
**Preferred option not available**		Metronidazole 500 mg tid 10 days	Vancomycin taper and pulse	Vancomycin taper and pulse
**Severe CDI**		Vancomycin or Fidaxomicin Oral administration not possible: local delivery +/- adjunctive therapy with i.v. metronidazole or i.v. tigecycline		
**Severe-complicated CDI and refractory severe complicated CDI**		Vancomycin or Fidaxomicin Multidisciplinary approach with surgical consultation Consider i.v. tigecycline and FMT when refractory		

The most important potential side effect is the risk of pathogen transmission and the possibility of developing contaminated small bowel syndrome in case of oral administration. Other possible complications include, depending on the method used, intestinal perforation caused by colonoscopy (the risk of which is increased by local inflammation) and, when using NG or NJ tubes, vomiting and subsequent aspiration, which may lead to pneumonia.

#### 1.2.3 Further treatment options

There are other ways to restore normal gut flora besides antibiotics or FMT. Toxins of *C. difficile* can be neutralized by monoclonal antibodies (bezlotoxumab, actoxumab (Džunková et al., 2016; Wilcox et al., 2017; Gerding et al., 2018; Prabhu et al., 2018; Salavert et al., 2018; Chen et al., 2021)), while re-establishment of a healthy microenvironment can be attempted through inoculation of certain bacterial strains (SER-109) in a similar way to FMT (McGovern et al., 2021; Feuerstadt et al., 2022). Bacteriophage therapy can also be used to target the bacterium directly (Mondal et al., 2020; Selle et al., 2020; Whittle et al., 2022). The first FDA approved fecal microbiota product (RBX2660, “Rebyota”) has shown success rates above 70% (Orenstein et al., 2016; Khanna et al., 2022; Orenstein et al., 2022). This product is made of human stool and administered as an enema. It is stored in form of a suspension in a single-dose ready-to-use enema bag at -80°C. The product can be stored at room temperature for up to 2 days prior to administration (Orenstein et al., 2016).

## 2 Materials and methods

### 2.1 Study design

The main aim of this research was to develop an effective encapsulation protocol to improve the practical application of fecal transplantation. We investigated whether the administration of a bacteria-free (or significantly reduced bacterial count) formulation is sufficient in CDI. Lyophilized fecal preparation was filled into capsules and stored at -20°C until administration.

The cure rate was recorded after capsule administration and the recurrence rate at one-year follow-up.

In parallel with the clinical trial, we assessed bacterial survival rates in the lyophilizates by periodic inoculation.

### 2.2 Population

Patients treated for CDI at the Department of Infectious Diseases, 1st Department of Internal Medicine, University of Pécs Clinical Centre between January 2018 and December 2019 were included in the study. During this period, a total of 28 patients received FMT with capsules. The intervention was offered for recurrent CDI when at least two recurrences occurred within 1 year.

After signing the informed consent form, patients were randomly assigned to receive either the supernatant (group A: 16 patients) or the sediment (group B: 12 patients).

The mean age of group A was 64.79 years, 10 women and 6 men. Group B had an average age of 66.47 years, 7 women and 5 men. The median number of previous relapses was 2 in both groups. **Table 2** summarizes the baseline characteristics of the two groups.

**Table 2:** The baseline characteristics of group A and B.

	Female/male/Sum	Mean age	Previous recurrences (median)
Group A	10/6/16	64,79 years	2
Group B	7/5/12	66,47 years	2

### 2.3 Donor criteria

Stool samples were provided by one donor throughout the clinical trial. Donor selection was based on criteria in line with international recommendations and was done by direct recruitment. The selection criteria were as follows:

Age should be between 18 and 65 years, BMI below 30 kg/m^2^, there should be no moderate or severe malnutrition. No chronic internal medicine, immunologic, neuropsychiatric or oncologic disease. No transmissible infectious diseases and no therapy influencing the immune system and the gut microbiome.

The donor was a tall, thin, Caucasian man in his twenties with a normal BMI, on a normal diet, not a healthcare worker. After evaluation of a risk assessment questionnaire and protocol-based screening, we provided him with the necessary information and tools for appropriate sample collection. The clinical trial was conducted before the first confirmed European appearance of the SARS-Cov-2 virus and therefore no screening was performed in this direction. Laboratory screening tests are summarized in **Table 3**.

**Table 3:** Laboratory screening tests for donors.

	From blood	From stool	From perianal swab
**Bacteria**	*- Treponema pallidum subspecies pallidum* - enzyme immunoassay	- Enteral pathogen culture: *Salmonella, Shigella, Campylobacter* spp.*, Yersinia enterocolitica* - *Helicobacter pylori* - enzyme immunoassay	- *Vancomycin resistant Enterococcus* - antibiotic susceptibility test to detect poly-resistant strains - ESBL producing *Enterobacteriales* strains
	**From blood**	**From stool**	
**Viruses**	- Hepatitis A virus IgM – enzyme immunoassay - Hepatitis B virus surface antigen - enzyme immunoassay - Hepatitis C and E virus surface antibody - enzyme immunoassay - HIV 1 and 2 - enzyme immunoassay	- Norovirus - enzyme immunoassay or PCR - Rotavirus - enzyme immunoassay - ESBL and VRE – anuclease	
**Parasites**	- Entamoeba histolytica - enzyme immunoassay - Strongyloides stercoralis - enzyme immunoassay	- Parasites or their eggs - microscopic examination - Microsporidia - microscopic examination - Giardia antigen - enzyme immunoassay - Cryptosporidium - enzyme immunoassay - Isospora and Cyclospora - acid-fast staining	
**Others**	- Complete blood count - Liver function test - Sedimentation, C-reactive protein - HbA1c	- *C. difficile* toxin and glutamate dehydrogenase antigen detection	

### 2.4 Sample collection

The stool was collected at the donor’s home in a non-sterile, airtight specimen collection container and the specimen was transported at room temperature to the sample processing laboratory within 1 hour.

### 2.5 Sample preparation

Sample processing was supposed to start within 6 hours of defecation according to our protocol. A dedicated laminar suction box was used, which was disinfected with sporicidal disinfectant before each sample processing.

#### 2.5.1 Homogenization, separation

During this phase, 60 g of feces were homogenized in a household mixer in a container with a sealable lid in 200 ml of physiological (0.9%) saline solution at room temperature. The resulting suspension was filtered twice to remove the larger solids. Solids that were difficult to remove by mechanical filtration were removed by a short-time, low g centrifugation (MPW-380R, Poland) (10 min, 827 g, room temperature) using 50 ml centrifuge tubes (Sarstedt Inc. Nümbrecht, Germany). With this method, approximately 120 ml of macroscopically homogeneous suspension can be produced from a 60 g dose of feces. Subsequently, the sample was treated in 100 ml portions. According to our previous protocol, this is the volume that the patient would receive if injected by nasogastric tube; therefore, this was chosen as the initial volume. Samples prepared for capsule preparation were then subjected to further centrifugation, this time for 15 min at 3309 g in a centrifuge precooled to 4°C. This step resulted in a sediment of around 30 ml, to which 10 ml of physiological (0.9%) saline was added to facilitate further treatment. The supernatant (around 70 ml volume) was divided into two parts and the sediment (around 40 ml volume) was stored whole in 5 cm diameter glass containers at -20°C for 12 hours. Samples at -20°C were then lyophilized (Freeze Dryer Heto Drywinner model DW1.0) at -40°C under vacuum 4*10–4 mbar for 36 hours, and the lyophilized samples were homogenized in a porcelain mortar on room temperature.

#### 2.5.2 Lyophilization, encapsulation

The homogeneous lyophilizates were loaded into hard gelatin capsules of size “00” corresponding to the given volume using a capsulation device (Capsule Machine, Capsule Connection, LLC, Prescott AR, USA). From the initial volume of 100 ml, 4–6 capsules containing lyophilized sediment and 4–6 capsules containing lyophilized supernatant were prepared, depending on the dry matter content of the sample and the degree of compressibility during encapsulation. The capsules were then stored at -20°C until administration. The appearance of the lyophilized supernatant and sediment-filled capsules differed only in color shade. These capsules are water soluble and not resistant to gastric acid.

We also planned to test enterosolvent capsules (Vcaps^®^ Enteric Capsules, Capsugel, Cambridge, MA, USA) to protect the lyophilizates against gastric acid. These capsules were one size smaller, size “0”, resulting in a 45% increase in the number of capsules required to encapsulate a single dose of lyophilizate. Given the success of the normal capsules, enterosolvent capsules were ultimately not used in the clinical trial.

We also planned to encapsulate an unlyophilized fecal suspension. This required the creation of a water-insoluble coating on the inner wall of the capsules. We tried two formulations:

- Eudragit^®^ L 30 D-55 (Ph. Eur. Methacrylic Acid - Ethyl Acrylate Copolymer 187 (1:1) 30% dispersion).

- Eudragit^®^ RS 30 D (Ph. Eur. 0.3% Sodium Lauryl Sulphate, 1.2% 188 Polysorbate 80 - aqueous dispersion with 30% solids content).

Both dispersions deformed the capsules, which remained properly sealable (if not perfectly) after coating. The degree of deformation was found to be reduced by the use of a hair dryer. After contact with water, the pre-treated capsules retained their stiffness for 60 minutes for Eudragit^®^ L 30 D-55 and 5 minutes for Eudragit^®^ RS 30 D.

### 2.6 Administration

In patients scheduled for FMT, we stopped metronidazole or vancomycin treatment the day before the procedure and started proton pump inhibitor therapy (if not already ongoing) to raise pH of the gastric fluid and hence improve bacterial survival. We tried to achieve a more rapid intestinal transit of the drug and a reduction in the likelihood of vomiting by administering metoclopramide. Patients were not allowed to eat before taking the capsules on the day of transplantation. Patients were required to take 4–6 capsules, one at a time, in about 5 minutes. They were then not allowed to eat for 2 hours, during which time they had to remain in at least a semi-recumbent position.

### 2.7 Evaluation, follow up

Cure was defined as the resolution of clinical symptoms, relapse as the recurrence of symptoms within 6 months, and follow-up of patients was defined as one year.

### 2.8 *In vitro* survival of bacteria

Survival studies were performed on donor samples before and after lyophilization to determine how long samples could be stored using the presented method without loss of efficiency.

At the time of sample processing, a dilution series was prepared from 1–1 ml of the samples to be lyophilized (supernatant, sediment) and 1 ml of the fecal suspension before separation of the two fractions. Inoculation of these samples was performed immediately. At the end of lyophilization, volumes of around 0.01 g were measured from the lyophilization samples for immediate inoculation and for inoculation at 2 days, 1 week, 1 month, 3 months and 6 months. The measured samples were stored at 4 different temperatures: -80°C, -20°C, +4°C and +20°C (room temperature), i.e. 21–21 samples were taken from the two different lyophilizates. The samples were taken from 9 processed fecal samples (378 samples in total.) The samples to be inoculated were first diluted to the original concentration based on the masses measured before and after lyophilization, and then ten-fold serial dilutions were made. From the dilution series, a single loopful of inoculum was spread over the surface of the agar media and the media were incubated aerobically and anaerobically at 30°C.

One inoculation per dilution was prepared for each of the following media: blood agar (aerobic and anaerobic incubation), chocolate agar, chocolate agar with vancomycin, eosin-methylene blue agar, Sabouraud agar.

The census was carried out after 48 hours of incubation. Clearly infected media were excluded from the counts. All media where, despite higher dilution, multiple counts or colonies not complying with the dilution were detected were marked as clearly infected. Germ counts were expressed in CFU/ml (Colony Forming Unit).

## 3 Results

### 3.1 Capsule making workflow

The fundamental objective was to create an efficient encapsulation workflow. Using our protocol, an assistant can prepare a total of 4–6 doses of capsules within 72 hours, with a total of about 8 hours of work.

The most complex was to organize the ideal time to bring the donor stool to the lab, due to the time-consuming and time-critical nature of the initial steps. After 2–3 hours of preparation, the stool sample could be placed in the freezer overnight after the necessary centrifugation steps. The next morning, the lyophilization process could begin, lasting 36–48 hours. After lyophilization, approximately 2 hours were required to pulverize and encapsulate the lyophilizates.

### 3.2 Clinical results (sediment, supernatant)

The reference point for this clinical trial was the effectiveness of previous modalities of FMT. As expected, patients showed less aversion to the concept of taking capsules as opposed to taking them through different tubes. It should be noted, however, that in recurrent CDI in general, no significant persuasion was needed for the other modalities either.

In total, 28 cases of FMT with capsules were performed in the clinical trial. No anomalies were observed during their implementation; the most serious complication was mild abdominal discomfort.

In group A (where patients were given capsules containing lyophilized supernatant), 15 out of 16 patients recovered after a single dose of capsules (93.75%). Two of the recovered patients relapsed shortly after transplantation (within 3 weeks), but they were again asymptomatic after administration of fidaxomicin. (In the group of cured patients, one patient underwent colectomy for IBD.) During follow-up, one patient passed away due to a comorbidity unrelated to CDI. The history of the patient who did not respond to treatment prior to FMT included a total of 22 recurrences.

In group B (where patients were given capsules containing lyophilized sediment), 8 out of 12 patients recovered after the first dose (66.67%). None of them experienced a relapse, but one of them remained positive for *C. difficile* A toxin in stool without clinical symptoms. Two of the patients who did not recover did not respond to the rest of the therapeutic options, one of them recovered after another FMT and the other one finally recovered after treatment with fidaxomicin. One patient passed away during follow-up due to another disease.

The overall cure rate was 23/28 (82.14%). The results are summarized in **Table 4**.

**Table 4:** The clinical results with FMT capsules.

	Successful	Unsuccessful	Sum
Group A	15	1	16
Group B	8	4	12

### 3.3 Laboratory results (CFU)

The results of the inoculation experiments have already been reported in a previous publication (Varga et al., 2023).

Using concurrent aerobic and anaerobic cultures, we found a difference of approximately 600-fold between supernatant and sediment CFU counts. We did not investigate the possible contribution of spores to the residual bacterial counts in the supernatant. The results showed that the lyophilized supernatants did not contain any viable bacteria or bacterial spores after 6 months of storage at -20°C.

The experiment showed that survival was inversely related to storage temperature and storage time. Thus, survival was most favorable at -80°C, but the greatest difference was seen between samples kept at -20°C and +4°C. This suggests that storage at -20°C for 6 months seems to be adequate for survival.

## 4 Discussion

The presented clinical trial was conducted to improve the methodology of fecal transplantation. The described encapsulation workflow makes fecal transplantation significantly simpler and more flexible, providing a new tool for the investigation of other uses. The use of lyophilized, encapsulated stools avoids the use of nasogastric or nasojejunal tubes, thus reducing the risk of future clinical trial planning and making the intake of the capsules less burdensome for the patients. The planning of interventions is improved, and their constraints are reduced. These factors will hopefully lead to easier obtaining of the necessary licenses for trials. The capsulation protocol results in a longer sample preparation time than is required for the preparation of stool suspensions used in NG, NJ, or colonoscopic administration. However, the prepared capsules can be easily and reliably stored in a -20°C freezer, even for long periods of time. Due to the small volume, FMT can be performed immediately after taking the capsules out of the freezer.

As the inoculations during sample processing showed, the lyophilized supernatant-filled capsules were not nearly bacteria-free. However, they had significantly lower bacterial counts than those containing lyophilized sediment or the whole feces administered according to the previous procedure. A report has also shown that bacteria-free stool preparations produced by mechanical filtration can be used effectively (Ott et al., 2017), suggesting that it is not the bacteria or their spores that are administered, but rather the smaller size components of the stool that may be responsible for the effect. The inoculation results also showed that after 6 months, although the lyophilized supernatants did not contain viable bacteria or bacterial spores, they were still effective in CDI. These bacterial survival results provide a clue as to how much less viable bacteria may have been present in capsules used 1–2, or even 6 months later (stored at -20°C until then) than in freshly used capsules. In contrast, results with bacterial concentrates of known composition suggest that the administration of some strains is effective (McGovern et al., 2021; Feuerstadt et al., 2022). It should be noted that these strains were not obtained by culture but by purification of donor stool, for example with ethanol.

For the bacteria and fungi that can grow on the media we used, we obtained information on some of the microorganisms introduced during the FMT, but we did not investigate the quantity and diversity of viruses at all. Several new viruses have been discovered in fecal samples using metagenomic studies (Xie et al., 2013), but even before this, a bewilderingly high diversity was to be taken into account.

To date, none of the current therapeutic recommendations mention stool transplantation as a first-line treatment for CDI or any other disease. Although it is already routinely used in the treatment of recurrent CDI in several centers, it is unlikely to be the first-line treatment of choice in this disease in the future.

The complexity of the sample to be administered always carries the risk of iatrogenic contamination, as has unfortunately been the case in recent years (DeFilipp et al., 2019; Zellmer et al., 2021). Normally, such a risk does not apply to antibiotics, as the manufacturer can control exactly what is included in the product. Bacterial concentrates of known composition are halfway between the two approaches (Orenstein et al., 2016; McGovern et al., 2021; Feuerstadt et al., 2022; Khanna et al., 2022; Orenstein et al., 2022).

The efficacy of bacteria-free formulations highlights what is probably the most important grey area at present. If a proportion of CDI infections can be cured with a bacteria-free stool preparation, the reason for the failure of bacterial concentrates may be the lack of, or insufficient quantity of a component that has been killed, denatured or diluted in the preparation of the concentrates. This component is naturally present in all feces, so it is likely that fecal transfusions will continue to be part of the protocol. However, in some cases, neither antibiotics nor the use of whole feces has led to cure. The existence of this group may be the result of inappropriate donor selection but is certainly a result of the lack of information available on the microbiome.

Our research suggests that fecal transplantation should be performed as shown in **Table 5**.

**Table 5:** Suggested faecal transplantation protocol.

**Preparations**	Regularly request stool samples from eligible donors who have undergone the necessary screening tests, depending on the expected use.
	Stool sample processing should begin within 6 hours of defecation.
	Process the stool sample according to the methodology described in this publication.
	Use ‘00’ size hard gelatin capsules without protective coating can be used appropriately.
	Store lyophilized samples at -20°C until use (samples stored at -20°C can be used with adequate efficacy within 6 months.)
**Administration**	Discontinue metronidazole or vancomycin the day before the procedure.
	Start proton pump inhibitor therapy the day before the procedure.
	Administer metoclopramide to increase intestinal motility and reduce the risk of vomiting.
	Patients should not eat before taking capsules on the day of transplantation.
	Patients should not eat until at least 2 hours after taking the capsules, during which time they should remain in at least a semi-recumbent position.
**Follow up**	6-month follow-up after a successful procedure.

*C. difficile* infection is the only disease for which fecal transplantation is currently routinely used, so most of the information comes from this field. Clinical trials are also being carried out in other intestinal diseases, such as ulcerative colitis (CU) and Crohn’s disease. A significant number of cases have been reported in several places, but the evidence is still awaited. Based on individual case reports (Blanchaert et al., 2019; Costello et al., 2019; Crothers et al., 2021), as we have also seen in one of the patients, FMT in CU may improve the patient’s condition. However, it should be kept in mind that these are all individual observations. Patients received transplantation for essentially different indications, so the results should be interpreted with caution.

The lack of evidence combined with the cumbersome nature of the commonly used FMT methodology hampers further research in this field. It is anticipated that the use of capsules will remove a number of current barriers, both in terms of patients (burdensome intervention, aversions), approval (risks of intervention) and infrastructure (equipment, time and space requirements).

## Data availability statement

The raw data supporting the conclusions of this article will be made available by the authors, without undue reservation.

## Ethics statement

The studies involving human participants were reviewed and approved by Hungarian Medical Research Council (ETT-TUKEB), Budapest, Hungary. Approval number: IF-7762-4/2015. Local ethical approvement number: 7083 – PTE 2018. The patients/participants provided their written informed consent to participate in this study. Written informed consent was obtained from the individual(s) for the publication of any potentially identifiable images or data included in this article. 

## Author contributions

DS: Conceptualization, Investigation, Methodology, Writing – original draft, Writing – review & editing. AV: Methodology, Project administration, Writing – review & editing. ÁK: Investigation, Writing – review & editing. BH-K: Investigation, Writing – review & editing. PK: Conceptualization, Writing – review & editing. SP: Conceptualization, Writing – review & editing. BK: Conceptualization, Methodology, Writing – review & editing. ZP: Conceptualization, Investigation, Methodology, Project administration, Supervision, Writing – review & editing.
